# Relation of the average interaction field with the coercive and interaction field distributions in First order reversal curve diagrams of nanowire arrays

**DOI:** 10.1038/s41598-020-78279-1

**Published:** 2020-12-07

**Authors:** Y. G. Velázquez, A. Lobo Guerrero, J. M. Martínez, E. Araujo, M. R. Tabasum, B. Nysten, L. Piraux, A. Encinas

**Affiliations:** 1 Departamento de Matemáticas y Física, Instituto Tecnológico y de Estudios Superiores de Occidente, Periférico Sur Manuel Gómez Morín 8585, 45604 Tlaquepaque, Jalisco Mexico; 2 Área Académica de Ciencias de la Tierra y Materiales, Universidad Autónoma del Estado de Hidalgo, Carretera Pachuca Tulancingo km 4.5, Ciudad del Conocimiento, Mineral de la Reforma, Hidalgo, Mexico; 3 Escuela de Ingenieria y Ciencias, Tecnologico de Monterrey, Puebla, Atlixcáyotl 5718, Reserva Territorial Atlixcáyotl, 72453 Puebla, Pue. Mexico; 4 Institute of Condensed Matter and Nanosciences, Université Catholique de Louvain, Place Croix du Sud 1, 1348 Louvain-la-Neuve, Belgium; 5 División de Materiales Avanzados, Instituto Potosino de Investigación Científica y Tecnológica, Camino a la Presa de San José 2055, 78216 San Luis Potosí, SLP Mexico

**Keywords:** Materials science, Condensed-matter physics, Magnetic properties and materials

## Abstract

First-order reversal curve diagrams, or FORC diagrams, have been studied to determine if the widths of their distributions along the interaction and coercivity axes can be related to the mean-field magnetization dependent interaction field (MDIF). Arrays of nanowires with diameters ranging from 18 up to 100 nm and packing fractions varying from 0.4 to 12% have been analyzed. The mean-field MDIF has been measured using the remanence curves and used as a measuring scale on the FORC diagrams. Based on these measurements, the full width of the interaction field distribution and the full width at half maximum (FWHM) of the FORC distribution profile along the interaction field direction are shown to be proportional to the MDIF, and the relation between them is found. Moreover, by interpreting the full width of the coercive field distribution in terms of the dipolar induced shearing, a simple relation is found between the width of this distribution and the MDIF. Furthermore, we show that the width of the FORC distribution along the coercive field axis is equal to the width of the switching field distribution obtained by the derivation of the DC remanence curve. This was further verified with the switching field distribution determined using in-field magnetic force microscopy (MFM) for very low density nanowires. The results are further supported by the good agreement found between the experiments and the values calculated using the mean-field model, which provides analytical expressions for both FORC distributions.

## Introduction

First order reversal curves (FORC) diagrams are a measuring protocol introduced by Pike et al.^1^ that has received a considerable attention and is widely employed for the characterization of the magnetic properties of discrete magnetic materials such as geomagnetic samples^2,3^, nanowires^4–18^, nanopillars^12,19^, patterned materials^20^, recording media^21,22^, nanotubes^12^, dots^23^ and antidots^12,24,25^.

A FORC diagram corresponds to the contour plot of the FORC distribution $\rho (H_u,H_c)$ represented in the interaction and coercivity plane, $H_u$ and $H_c$, respectively. These diagrams are attractive since the FORC distribution is expected to provide detailed information of the interaction field and its effects in particle assemblies^1^ and under some circumstances, they allow to reconstruct the intrinsic properties of fine particle systems^26^. In non-interacting particle assemblies, the FORC diagram shows a single narrow distribution, or ridge, along the $H_c$ axis and in this case, it represents the intrinsic coercive field distribution (CFD)^14,19,27,28^. However, in assemblies of interacting particles, the interaction field modifies the width of the CFD, hereafter referred to as $\Delta _{\mathrm {CFD}}$, and leads to a second ridge along the $H_u$ axis, known as the interaction field distribution (IFD), whose width, $\Delta _{\mathrm {IFD}}$, has been shown to vary with the strength of the interaction field^1,19,26,27,29,30^.

Ideally, a FORC diagram should allow us to derive a quantitative measurement of the interaction field, provide information to interpret the width of the CFD, and eventually relate it to the intrinsic CFD. In practice, this has proven to be very difficult, and in many cases, only qualitative information is obtained from these diagrams. In consequence, the analysis, interpretation, and understanding of these diagrams is typically done by developing models to describe them^1,14,19,20,26,27,29–31^.

In the present study, FORC diagrams measured in arrays of nanowires (NW) have been analyzed considering a first-order mean-field approach for the dipolar interaction field and the shearing of the *M*(*H*) curves caused by the interaction. In particular, we assume a simple mean field approximation for the interaction field and that this average interaction field results solely in a dipolar shearing of the *M*(*H*) curves. Arrays of magnetic NWs are a model system due to their cylindrical shape and the easy to control their height as well as the interaction field strength^32,33^. Moreover, they have been shown to be well suited to be used for the analysis of FORC diagrams^14^. Our goal was twofold. First, to determine how much of the main features in the FORC diagrams can be reasonably well understood in terms of the simplest mean-field approximation for the interaction field and the shearing it induces on the magnetization curves. Secondly, to determine if with proper identification of these features, it is possible to quantify the value of the interaction field from the FORC diagrams. To do this, over 20 NW samples have been studied in order to obtain a clearer picture considering FORC diagrams with different characteristics. To this end, NWs with diameters ranging from 18 up to 100 nm and packing fractions between 0.4 and 12% have been considered. In all cases, the average value of the dipolar interaction field was determined independently from the isothermal (IRM) and DC demagnetizing (DCD) remanence curves^34^. This measured value of the interaction field has been used as a measuring scale in the FORC diagrams. In particular, we found that the width of the distributions along the interaction and coercive field axes scale linearly with the average interaction field value.

Moreover, the quantity $\Delta H_u$ introduced by Berón et al.^7^ that corresponds to the full width at half maximum (FWHM) of the FORC profile along the interaction field axis provides a measure of the interaction field, while at the same time corresponds to 1/3 of the full width of the distribution along the interaction field axis. Finally, the width of the FORC distribution along the coercive field axis is shown to scale with the MDIF, and its full width is shown to be equal to the full width of the switching field distribution (SFD) obtained as the derivative of the DCD remanence curve. A mean-field model for magnetization dependent interaction field^35^ has been used to interpret this relation, which has been further verified by comparing with the experimental results.

## Results

FORC diagrams have been measured in arrays of nanowires (NWs) made of Ni, NiFe, CoFe and Co, with diameters ranging from 18 up to 100 nm. The strength of the interaction field has been varied by considering arrays with packing fractions, *P*, as low as 0.4%, and as high as 12%, as described in the methods section.

**Figure 1:**
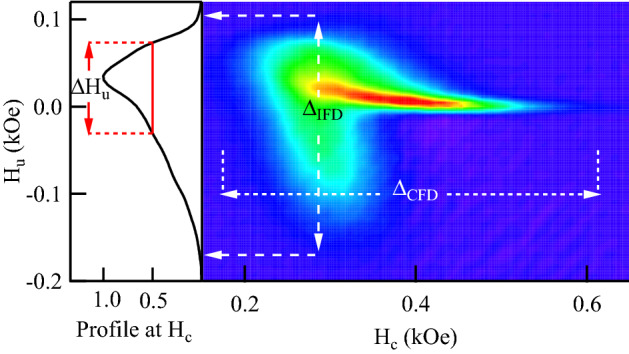
FORC diagram plotted in the $H_c, H_u$ plane measured for the sample S17 (Ni, $\phi$=100nm, *P*=2.0%), where the measured quantities are indicated: the interaction field distribution $\Delta _{\mathrm {IFD}}$, the coercive field distribution $\Delta _{\mathrm {CFD}}$ and $\Delta H_u$.

Figure 1 shows a typical FORC diagram measured for the sample S17 (Ni, $\phi$=100nm, *P*=2.0%), where both $\Delta _{\mathrm {IFD}}$ and $\Delta _{\mathrm {CFD}}$ ridges can be observed. As indicated in the figure, there are three quantities that have been obtained from each diagram and used for the present study. The first quantity corresponds to the full width of the interaction field distribution $\Delta _{\mathrm {IFD}}$, which was taken as the maximum span of the distribution along the $H_u$ axis. The second, named $\Delta H_u$, corresponds to the full width at half maximum (FWHM) of the FORC profile along the interaction field axis at the field value $H_c$ where $\Delta _{\mathrm {IFD}}$ was found^7^. Finally, the full width of the coercive field distribution $\Delta _{\mathrm {CFD}}$ that was measured as the maximum span of the distribution along the $H_c$ axis. In the following, the relation between these quantities with the interaction field as well as among them is shown. The FORC diagrams for the 22 samples presented in this study with their corresponding measured quantities ($\Delta _{\mathrm {IFD}}$ and $\Delta _{\mathrm {CFD}}$) are shown in Supplementary Fig. SI1.

### The interaction field distribution

Since their introduction by Pike et al.^1^ FORC diagrams have been considered as an experimental method to measure interaction effects in particle assemblies. However, the quantitative capabilities of the FORC diagrams remain unclear. On the one hand, the full width of the interaction field distribution, $\Delta _{\mathrm {IFD}}$, is known to scale with the mean-field interaction field value^19^, which is supported by a large number of qualitative experimental as well as numerical results. Yet, no expressions are known that relate the interaction field strength with $\Delta _{\mathrm {IFD}}$. Moreover, Béron et al.^7^ have proposed that $\Delta H_u$ corresponds to the magnetostatic (dipolar) interaction at saturation. However, as seen in Fig. 1, $\Delta _{\mathrm {IFD}}>\Delta H_u$ and therefore, it is interesting to determine a relation between these quantities and the interaction field.

To analyze the role of the dipolar interaction in the FORC diagrams, the interaction field in NW arrays has been measured using the IRM and DCD remanence curves (hereafter $\alpha _{z}^{E}$, where the superscript *E* implies that it is an experimentally measured value)^35^, given by Eq. (12), as described in the methods section. This method is independent of the FORC diagrams, so it provides a measuring scale to compare and analyze the effects of the interaction field on these diagrams and their relation with $\Delta _{\mathrm {IFD}}$ and $\Delta H_u$. Furthermore, the interaction field determined using the IRM and DCD remanence curves ($\alpha _{z}^{E}$), corresponds to the axial component of the magnetization dependent average interaction field derived from a mean field model (hereafter $\alpha _{z}^{T}$, where the superscript *T* implies that it is a theoretical value), namely^34^

1 $$\begin{aligned} \alpha _z^T=2 \pi M_s P \end{aligned}$$

This expression follows from the mean field model for a 2D NW array. Where the effective demagnetizing field of a NW contains two contributions, the self demagnetizing field and the dipolar interaction field which is the field experienced by the wire due to the stray field of the rest of the wires in the array. In particular, Eq. (1) is the magnetiztion dependent interaction field coefficient of the axial component of the effective demagnetizing field for a NW array, namely $H_{Dz}^{eff}= 2\pi M_s P(1+m)$, or $H_{Dz}^{eff}= \alpha _z^T (1+m)$^34^.

**Figure 2:**
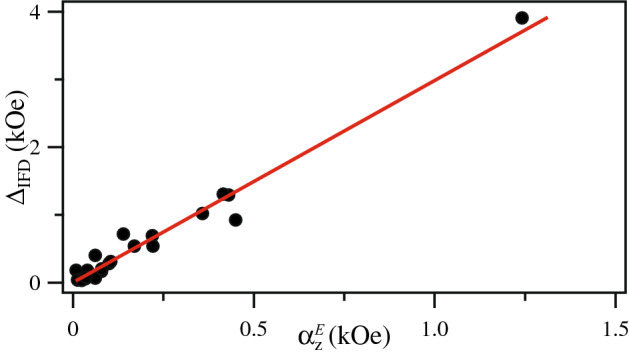
Measured width of the IFD distribution, $\Delta _{\mathrm {IFD}}$, plotted as a function of the axial component of the interaction field $\alpha _z^{E}$ measured from the IRM/DCD remanence curves. The straight line corresponds to the fit of $\Delta _{\mathrm {IFD}}=3\alpha _z$.

Regarding the full width of the IFD in the FORC diagrams, the measured $\Delta _{\mathrm {IFD}}$ values have been plotted against the measured component of the interaction field $\alpha _z^E$, and the results are shown in Fig. 2. As seen in this figure, the entire data set can be very well fitted to a straight line with a slope equal to 3. Thus showing a clear relation between $\Delta _{\mathrm {IFD}}$ and $\alpha _z$. From these results and Eq. (1), it follows that

2 $$\begin{aligned} \Delta _{\mathrm {IFD}} = 3\alpha _z =6\pi M_sP \end{aligned}$$

which shows that the full width of the IFD distribution is related to the strength of the interaction field. The equivalent figure with the theoretical interaction field ($\alpha _{z}^{T}$) is shown in Supplementary Fig. SI2, for which a good agreement is also found.

For $\Delta H_u$, which was proposed as the total interaction field at saturation^7^, we now show that this quantity does provide a value of the interaction field, and how it relates to the width of the IFD distribution. $\Delta H_u$ was related to the interaction field at saturation^7^, and it refers to the maximum value attained by the interaction field when the system is saturated. This is if we consider the interaction field such that $H_{int}=\alpha m$, then $\alpha$ is the interaction field at saturation ($m=1$). In this sense, Béron et al.^7^ consider the interaction field at saturation as the linear sum of two contributions, the average ($\alpha$) and the local ($2\sigma _{int}$) interaction fields,

3 $$\begin{aligned} H_{int-sat}= 2\sigma _{int} + \alpha \end{aligned}$$

This is done to account for the fact that a realistic description of a particulate assembly should consider that the interaction field felt by each particle is different. However, $\Delta H_u$ is a single value measurement of the interaction field of the entire particle assembly, and thus it must be interpreted as an average value. In this case, $2\sigma _{int}$=0, and it is implicitly assumed that every particle is subjected to the same interaction field.

First, we compare values of $\Delta H_u$ measured in previous studies with the axial component of the magnetization dependent interaction, Eq. (1). To this end, $\alpha _{z}^{T}$ has been calculated as a function of the NWs diameter ($\phi$) while keeping the center to center distance between them (*D*) constant using the expression for the packing fraction of a 2D hexagonal array, $P=(\pi \phi ^2)/(2\sqrt{3}D^2)$, this is,

4 $$\begin{aligned} \alpha _z^{T}= \frac{\pi ^2 M_s}{\sqrt{3}D^2}\phi ^2 \end{aligned}$$

**Figure 3:**
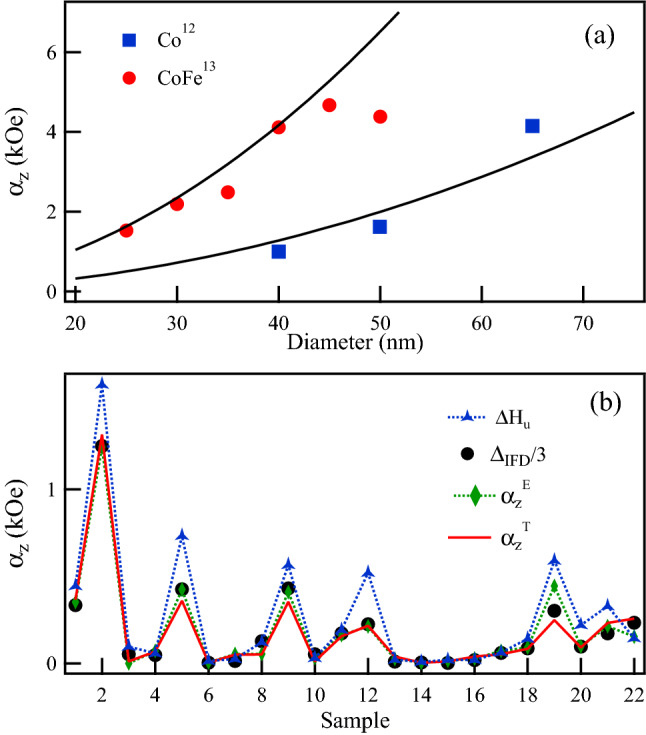
Interaction field coefficient, $\alpha _z$ (**a**) calculated using Eq. (4) for Co and CoFe NWs (continuous lines) compared to the experimental values of $\Delta H_u$ reported in Refs.^12,13^, and (**b**) Comparison of the values of $\Delta H_u$ and $\Delta _{\mathrm {IFD}}/3$ measured from the FORC diagrams and the experimental ($\alpha _z^E$) as well as theoretical ($\alpha _z^T$) values of the axial component of the magnetization dependent interaction field.

This expression has been calculated and compared with the values of $\Delta H_u$ reported for Co ($M_s$=1400 emu/cm$^3$ and $D=100$ nm)^12^ and CoFe ($M_s$=1991.5 emu/cm$^3$ and $D=66$ nm)^13^ NWs grown in hexagonally ordered anodized alumina templates. The results are shown in Fig. 3a.

As seen from these results, the values determined using Eq. (4) are consistent with those reported for $\Delta H_u$ in both Co^12^ and CoFe^13^ NWs, suggesting that $\Delta H_u=\alpha _z$ and from the previous results, Eq. (2), it follows that

5 $$\begin{aligned} \Delta H_u=\frac{\Delta _{\mathrm {IFD}}}{3} \end{aligned}$$

To further corroborate this result, Fig.  3b compares the values of $\Delta H_u$, $\Delta _{\mathrm {IFD}}/3$ and $\alpha _z^E$ measured on all the samples listed in Table 1 along with the values, $\alpha _z^T$, calculated using Eq. (1). Overall, the results presented in Fig. 3b shows a good agreement, and they confirm that $\Delta H_u=\alpha _z=\Delta _{\mathrm {IFD}}/3$ that numerically corresponds to the axial component of the magnetization dependent average interaction field. Notice in this figure that the largest errors are obtained for $\Delta H_u$, which after a further analysis it was found to be related to the non-uniformity of the IFD profile in those samples with the highest packing fraction. Indeed, except sample S12 (*P*=4.5%), samples S2, S5, S9, and S19, have packing fractions $P\ge 10$%. Supplementary Fig. SI3 compares the interaction field distribution profile of samples with high packing fractions (S2, S5, S9, S12, and S19) and those with low packing fractions (S3, S6, S13, S14, S15). These results show that both $\Delta H_u$ and $\Delta _{\mathrm {IFD}}$ are related, and both provide a quantitative measurement of the interaction field, but they correspond to different forms of expressing it.

### The coercive field distribution

Another point of interest regarding the use of FORC diagrams is related to the interpretation of the measured distribution along the $H_c$ as the switching field distribution (SFD) and its relation to the intrinsic SFD. Previous work on nanopillars and NWs have provided some insight into this point. For example, model simulations done on 2D arrays of cylindrical nanopillars have shown that the width of the distribution measured along the $H_c$ axis in the FORC diagram ($\Delta _{\mathrm {CFD}}$) can be reproduced from simulations considering an intrinsic switching field distribution and a mean-field antiferromagnetic dipolar interaction^19^. In this sense, it is well known that a magnetization dependent antiferromagnetic mean-field interaction is also related to the dipolar shearing of the hysteresis loop and any other *M*(*H*) measurement^35^. In the case of FORC diagrams in NWs, model calculations reported previously also suggest that the width of the CFD could be related to the shearing of the hysteresis loop and the measured FORCs^14^. Indeed, in Fig. 12 of this work^14^, the authors relate the largest $H_c$ value of the CFD with the nanowire with the highest coercivity in the array.

On these bases, we have considered the measured $\Delta _{\mathrm {CFD}}$ in the FORC diagrams in terms of the shearing induced by the interaction field on the intrinsic SFD. In this sense, the average mean-field magnetization dependent interaction field can be written as $H_{int}=\alpha m$, where $\alpha$ is the interaction field coefficient, which for NW arrays is antiferromagnetic so $\alpha >0$ and $m \in [-1,1]$. In a mean-field analysis, the shearing of the *M*(*H*) curves results from the fact that the total field experienced by a given particle in an interacting assembly corresponds to the applied field and the field exerted on this particle by the rest. Moreover, as shown previously, the shearing of the *M*(*H*) loops measured along the wire axis is due to the component along the easy axis of the magnetization dependent interaction field coefficient, $\alpha _z$, Eq. (12)^35^.

In a NW array, the NW with the highest coercivity is the last wire to switch on the major hysteresis loop; therefore it is the one subjected to the largest interaction field, and it is also the one whose coercive fields shift the most when the hysteresis loop is sheared due to the interaction field. When the field is swept from positive to negative saturation, the last wire to switch its magnetization has a coercive field that is shifted by $\delta =\alpha _z$ to more negative field values. Inversely, when the field is swept from negative to positive saturation, the last wire to switch has a coercive field shifted by the same quantity towards more positive fields. So for a given interaction field value, $\Delta _{\mathrm {CFD}}$ should increase its width with respect to the non-interacting case, by a quantity of $2 \alpha _z$. Moreover, this shearing is symmetric with respect to the coercive field $H_c$ measured on the major hysteresis loop. Then, $\Delta _{\mathrm {CFD}}$, can be written as

6 $$\begin{aligned} \Delta _{\mathrm {CFD}}= 2\alpha _z + H_c \end{aligned}$$

**Figure 4:**
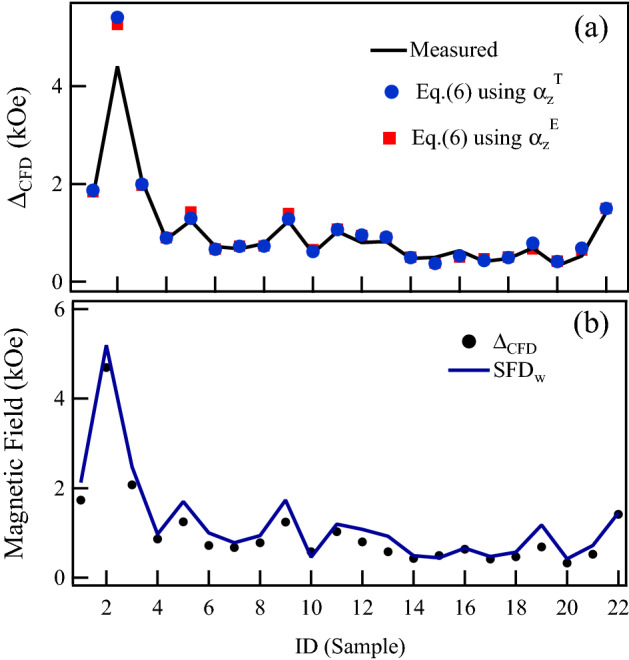
(**a**) Coercive field distribution width, $\Delta _{\mathrm {CFD}}$, measured from the FORC diagrams (continuous line) compared with the width calculated with Eq. (6) using the theoretical (circles) and experimental values (squares) of $\alpha _z$, and (**b**) Comparison between the full width of the FORC-CFD, $\Delta _{\mathrm {CFD}}$ and the full width of the SFD, $\hbox {SFD}_{\mathrm {W}}$ obtained by derivation of the major DCD remanence curve.

To test this relation, Fig. 4a compares $\Delta _{\mathrm {CFD}}$ measured from the FORC diagrams (continuous line) with the widths calculated with Eq. (6) using the theoretical (circles), Eq. (1), and experimental (squares) values of $\alpha _z$ for all the samples considered in this study. These results show an excellent agreement that further supports the interpretation of the dipolar induced shearing and broadening of the intrinsic coercive or switching field distribution as the main effect leading to the observed $\Delta _{\mathrm {CFD}}$.

The shearing of the *M*(*H*) curves also leads to the broadening of their derivatives, which correspond to the switching field distribution. In particular, for the major loop or the DCD, the measured SFD is interpreted as the intrinsic SFD broadened by the shearing of the *M*(*H*) curve. Therefore, the total width of the measured SFD scales with the interaction field as well as with $\Delta _{\mathrm {CFD}}$. Figure 4b compares the $\Delta _{\mathrm {CFD}}$ obtained from the FORC diagrams with the total width of the SFD ($\hbox {SFD}_{\mathrm {W}}$) obtained by direct derivation of the DCD remanence curve, as described in the methods section. As seen from the figure, a very good agreement is observed between these quantities, further supporting that the distribution along the coercive field axis in a FORC diagram corresponds to the intrinsic SFD broadened by the shearing of the return curves induced by the interaction field.

Finally, for an array of non-interacting particles, a measurement providing directly or indirectly, the SFD should yield the intrinsic SFD. In this sense, the CFD has been measured in samples with very low packing fractions, this is, in samples where no significant shearing due to the interaction field is present and thus, the intrinsic coercive (or switching) field distribution is not expected to be sensibly modified by the interaction field.

**Figure 5:**
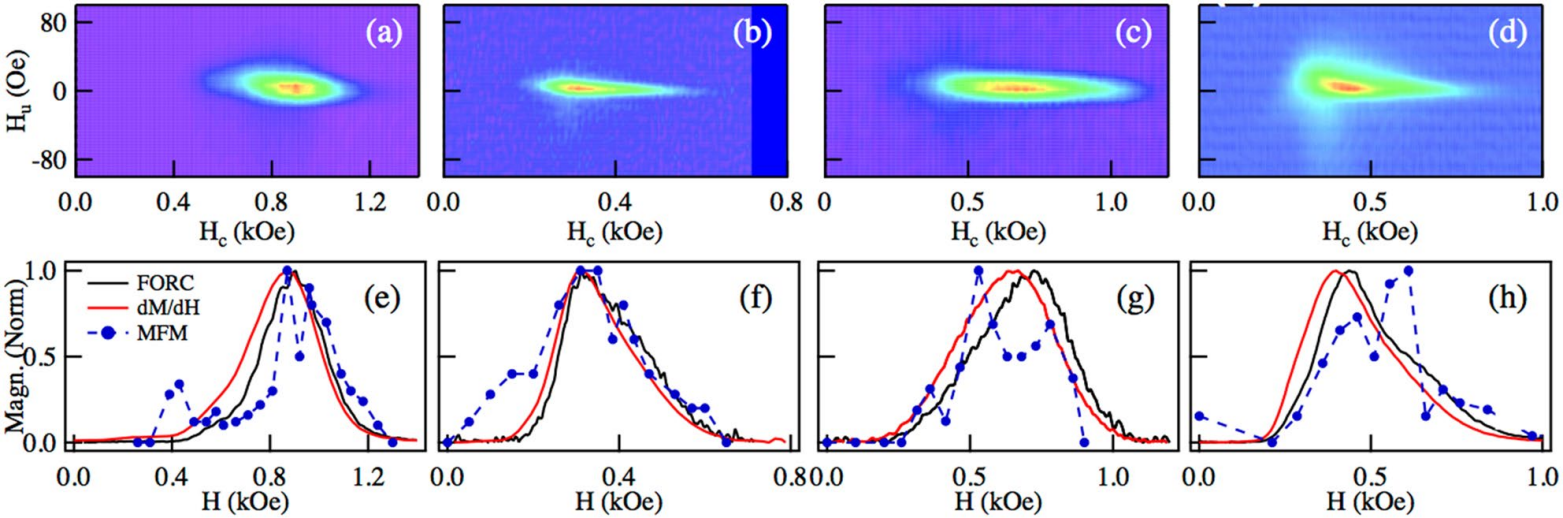
FORC diagram measured in low packing fraction ($P=$ 0.4%) NWs, (**a**) 50 nm CoFe [S13], (**b**) 71 nm NiFe [S15], (**c**) 50 nm Ni [S6], and (**d**) 71 nm CoFe [S16]. The corresponding CFDs measured using the FORC diagram, the SFD (*dM*/*dH*) and MFM are shown in (**e**)–(**h**), respectively.

Figure 5 shows the FORC diagram measured in NWs with low packing fraction ($P=$ 0.4%), (a) 50 nm CoFe [S13], (b) 71 nm NiFe [S15], (c) 50 nm Ni [S6], and (d) 71 nm CoFe [S16]. While (e)–(h) compare the corresponding CFD profiles measured from the FORC diagram, and the SFD obtained from the derivative of the DCD curve (*dM*/*dH*) and from the MFM measurements (as indicated in the methods section). As seen in the figure, the coercive or switching field distributions determined from these different measurements show a good agreement and allow them to state that they are equivalent. This suggests that in an assembly of non-interacting particles, the coercive or switching field distributions correspond to the intrinsic distribution of the assembly of particles. Moreover, since there is no shearing of the *M*(*H*) measurements due to the dipolar interaction, the measured distribution is independent of the measuring technique or protocol^36^.

## Discussion

The results presented above show a clear relation between the quantities measured from the FORC diagrams, namely $\Delta _{\mathrm {IFD}}$, $\Delta H_u$ and $\Delta _{\mathrm {CFD}}$, with the axial component of the magnetization dependent average interaction field ($\alpha _z$). These relations follow on few simple assumptions such as the mean-field average interaction field, and the shearing it produces on the *M*(*H*) curves. No attempts were made to relate our approach to a given model to seek for a more detailed interpretation. In this sense, the identification only serves to provide some insight into the main characteristics of the FORC diagrams in NW arrays and to provide guidelines to quantify the average value of the interaction field from them. However, the results are consistent with a recent report by Ruta et al.^37^ who concluded that in systems with no collective effects due to strong interaction effects, can be treated using the mean-field approach based on the average value of the interaction field.

The relation found between $\Delta H_u$ and $\alpha _z$, means that for arrays of NWs [Eq. (1)],

7 $$\begin{aligned} \Delta H_u = 2\pi M_sP \end{aligned}$$

This has been verified by the calculations done using Eq. (4) and the results are shown in Fig. 3. This relation is important since it is sometimes assumed that the interaction field in NWs is $H_{int}=4\pi M_s P$^38^. However, the relation between these two values has been explained^35^.

The CFD width has been analyzed on the basis that it corresponds to an intrinsic CFD, which is broadened by the shearing of the *M*(*H*) curves due to the dipolar interaction field. An empirical expression has been used to show that the $\Delta _{\mathrm {CFD}}$ does, in fact, scale with $\alpha _z$, which is responsible for the interaction induced shearing. This was also confirmed by showing measurements done with different methods, that in the non-interacting limit, the FORC distribution corresponds to the intrinsic CFD. Analyzing the measured CFD from a mean-field perspective, it follows that the intrinsic coercivity of individual NWs is only shifted and not modified by the interaction field. Compared with the width of the CFD of the non-interacting particle assembly, the observed width of the CFD are larger if the interaction field is antiferromagnetic, or smaller for the case of a ferromagnetic interaction and the observed coercivities do not correspond to the intrinsic ones^20^. Since the $\Delta _{\mathrm {CFD}}$ depends on the strength of the interaction field and the coercive field measured on the hysteresis loop, Eq. (6), the dipolar interaction can also be determined from the width of the CFD. Moreover, since Eq. (6) relates $\alpha _z$ with $\Delta _{\mathrm {CFD}}$ and the coercive field, then from the measurement of $\Delta _{\mathrm {CFD}}$ in the FORC diagram and $H_c$ its possible to determine the component of the magnetization dependent interaction field, $\alpha _z$, as

8 $$\begin{aligned} \alpha _z=\frac{1}{2}(\Delta _{\mathrm {CFD}} - H_c) \end{aligned}$$

The results are shown in Supplementary Fig. SI4 and they are compared with the experimental and theoretical values. Overall a good agreement is observed thus providing support to the fact that the interaction field can also be quantified from the FORC diagrams using $\Delta _{\mathrm {CFD}}$.

Finally using the Eqs. (2) and (6) the following relation between $\Delta _{\mathrm {IFD}}$ and $\Delta _{\mathrm {CFD}}$ is obtained,

9 $$\begin{aligned} \Delta _{\mathrm {IFD}}= \frac{3}{2} \left( \Delta _{\mathrm {CFD}}-H_c \right) \end{aligned}$$

**Figure 6:**
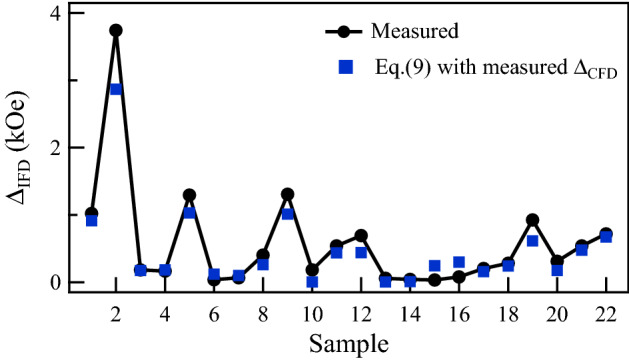
Interaction field distribution width, $\Delta _{\mathrm {IFD}}$, measured from the FORC diagrams (line with circles) compared with the width calculated with Eq. (9) using the measured values of $\Delta _{\mathrm {CFD}}$ and $H_c$ (squares).

This relation is based only on the width of the two distributions obtained from the FORC diagrams and the coercive field measured in the hysteresis loop. Figure 6 compares the measured $\Delta _{\mathrm {IFD}}$ with the values obtained using Eq. (9) with $H_c$ measured from the major hysteresis loop and the measured values of $\Delta _{\mathrm {CFD}}$. As seen in the figure, an overall agreement is observed for all the samples. A consequence of these results is that the sample coercivity, or the coercive field measured from the major hysteresis loop can be expressed in terms of these two distributions,

10 $$\begin{aligned} H_c=\Delta _{\mathrm {CFD}}-\frac{2}{3}\Delta _{\mathrm {IFD}} \end{aligned}$$

Therefore, if $\mathrm {CFD}$ and the SFD increase or are broadened by the interaction field, then they both scale proportionally to the strength of the interaction field. Finally, it is worth mentioning that the main relations found between the width of the distributions in the FORC diagrams and the interaction field, as well as with the width of the SFD, rely on measurements of the full width of the distributions. In practice, this is a difficult problem because there is no absolute or unique criteria to measure the full width of a measured distribution. The exact shape and extent of the tails in these distributions are related to the arrival to saturation, which can vary significantly from sample to sample. On the other hand, using other quantities to quantify the width of the distributions, such as the full width at half maximum, are also limited by the shape or complexity of the distribution. These results, supported by the good agreement found with the model, show that the FORC diagrams can serve to quantify the value of the dipolar interaction using $\Delta _{\mathrm {IFD}}$, $\Delta _{\mathrm {CFD}}$ or $\Delta H_u$. Furthermore, from either one of these quantities, the packing fraction can be determined if the value of $M_s$ is known.

## Conclusions

In conclusion, using nanowire arrays, we have found a clear relation between the average value of the dipolar interaction field and the width of the distributions along the interaction and coercivity axes in FORC diagrams. Using a mean-field analysis and considering the interaction field-induced shearing of the magnetization curves, we show that the full width of the FORC distributions is proportional to the axial component of the magnetization dependent interaction field. Moreover, simple relations between these quantities and the FWHM of the FORC profile along the interaction axis have been validated. Therefore, the three quantities measured in a FORC diagram, namely the width of the distributions along the interaction and coercivity axes as well as the FWHM of the FORC profile along the interaction axis, provide a quantitative measure of the axial component of the magnetization dependent interaction field. Furthermore, we have also shown that the full-width of the coercive field distribution in the FORC diagram is equal to the full width of the switching field distribution (SFD) and that both are proportional to the interaction field. Our results represent an approximative but effective and practical approach using FORC diagrams to obtain quantitative values of the interaction field and the width of the SFD in nanowire arrays and other 2D particle assemblies with perpendicular anisotropy.

## Methods

### Sample preparation

Arrays of Ni, Co, NiFe and CoFe nanowires have been grown by electrodeposition into nanoporous polycarbonate (PC) and anodized alumina axide (AAO) membranes. These 21 $\mu$m thick track-etched PC membranes (from it4ip S. A.) have the pores parallel to each other but randomly distributed and characterized by their average packing fraction (*P*) or porosity that is defined as the product of the pore density and the area of a single pore. These membranes have improved pore orientation, shape, size distribution and surface roughness^39^. The 90 $\mu$m thick AAO membranes (from Synkera) have hexagonally ordered parallel pores and the packing fraction is given in terms of the pore diameter ($\phi$) and the interpore distance (*D*).

For the electrodeposition, a Cr/Au layer evaporated previously on one side of the membrane serves as a cathode. Depositions were done at room temperature with a constant potential using an Ag/AgCl reference electrode and a Pt counter electrode. For CoFe a 40 g/l $\hbox {FeSO}_4$ +80 g/l $\hbox {CoSO}_4$ + 30 g/l $\hbox {H}_3\hbox {BO}_3$ electrolyte was used with a potential of V = – 0.9 V, while for NiFe the electrolyte contained 5.56 g/l $\hbox {FeSO}_4$ + 131.42 g/l $\hbox {NiSO}_4$ + 30 g/l $\hbox {H}_3\hbox {BO}_3$ and deposition done at V = – 1.1 V. For Ni NWs, the composition of the electrolyte was 119.38 g/l $\hbox {NiSO}_4$ and 30.91 g/l $\hbox {H}_3\hbox {BO}_3$. Cobalt nanowires have been grown at V = – 1 V using a 238.5 g/l $\hbox {CoSO}_4$ + 30 g/l $\hbox {H}_3\hbox {BO}_3$ electrolyte with the pH set to 2.0 by addition of $\hbox {H}_2\hbox {SO}_4$ to favor a non-textured polycrystalline fcc-like Co structure^40^. Full details of the preparation method can be found elsewhere^32,41^. For all samples, the wire length has been kept between 18 and 20 $\mu$m, so the NW aspect ratio (height/diameter) remains between 200 and 1000. Moreover, these materials have a non-textured polycrystalline cubic structure so that the magnetocrystalline anisotropy contribution can be neglected. Table 1 shows the details of the 22 samples considered in this study.

To clarify the basic geometry of these two dimensional NW arrays, Supplementary Fig. SI5 shows the top view SEM micrographs of three PC membranes. One (a) with the lowest porosity used, [S14] with 71 nm diameter and 0.4% porosity and two with high porosities (b) [S19] with 100 nm diameter and 10% porosity and (c) [S9] with 50 nm diameter and 11.8% porosity.

**Table 1:** List of characterized samples. For each sample the following quantities are: sample number, material, the pore diameter $\varphi$, the packing fraction *P*, the coercive field $H_c$, theoretical value of the interaction field $\alpha _z^T$, and the dipolar interaction coefficient $\alpha _z^E$ obtained from the IRM and DCD remanence curves.

	Material	$\varphi$ (nm)	*P* (%)	$H_c$(Oe)	$\alpha ^T_z$ (Oe)	$\alpha ^E_z$(Oe)
S1	Ni	18	7.5	1126	371	358
S2	CoFe	18	11	2782	1313	1242
S3	NiFe	20	0.4	1956	19	9
S4	Ni	40	2.5	741	76	79
S5	Ni	40	12.0	564	366	430
S6	Ni	50	0.4	640	12	14
S7	Ni	50	1.9	606	58	61
S8	Ni	50	2.0	607	60	61
S9	Ni	50	11.8	566	359	415
S10	NiFe	50	0.4	578	19	39
S11	NiFe	55	3.3	737	163	169
S12	NiFe	50	4.5	507	222	219
S13	CoFe	50	0.4	827	47	34
S14	Ni	71	0.4	478	12	12
S15	NiFe	71	0.4	333	19	24
S16	CoFe	71	0.4	435	47	36
S17	Ni	100	2.0	310	61	79
S18	Ni	100	3.0	308	91	99
S19	Ni	100	10.0	279	255	450
S20	NiFe	100	2.0	211	99	105
S21	CoFe	100	2.0	206	239	221
S22	Co	30	3.0	970	264	161

### Magnetic measurements

Magnetometry measurements have been done at room temperature using an alternating gradient magnetometer with the field applied parallel to the NWs axes.

The FORC diagrams are calculated as^1^,

11 $$\begin{aligned} \rho (H_r,H)=-\frac{1}{2}\frac{\partial ^2M(H_r,H)}{\partial H_r\partial H} \end{aligned}$$

where *H* is the applied field and $H_r$ is the return field. The FORC diagram is plotted in the interaction-coercivity plane, whose coordinates are defined as $H_u=-(H+H_r)/2$ and $H_c=(H-H_r)/2$. For each sample, 180 FORCs were measured and they have been processed using the FORCinel software package^42^ and in all cases, the smoothing factor was kept at a value of 2.

Major hysteresis loops, as well as the DCD and IRM remanence curves, have been measured. The IRM curve is obtained by first demagnetizing the sample using an alternating magnetic field with decreasing amplitude. The initially demagnetized sample is subjected to a sequence of incremental steps of a positive magnetic field. Between each increment, the field is switched off and the corresponding remanent magnetization is measured, this is repeated until the positive saturated state is attained. The IRM curve is the plot of the measured remanence against the final field value of the corresponding field increment. For the DCD curve, the system is initially saturated with a large positive field and then this field is removed, so the initial state corresponds to the remanent state of the major hysteresis loop. The sample is then subjected to a sequence of increasing steps of a negative magnetic field which are done until the final value is large enough to saturate the sample in the negative saturation state. After each incremental step, the field is switched off and the remanent magnetization is measured. The DCD curve is the plot of the measured remanence against the final field value of the corresponding field increment.

**Figure 7:**
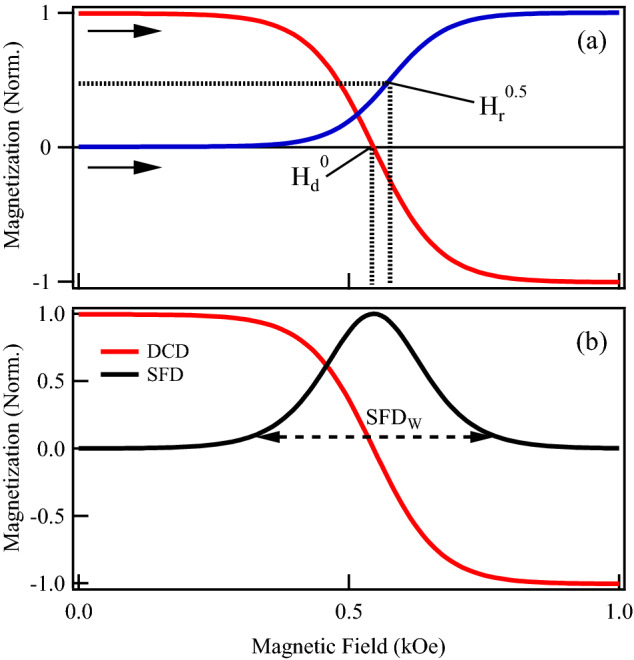
(**a**) Typical IRM and DCD remanence curves. The field value at which the normalized IRM curve is equal to 0.5, $H_r^{0.5}$, and the field value at which the DCD curve is zero $H_d^0$ are indicated. The arrows indicate the field sweep sense. (**b**) The DCD remanence curve and its derivative (SFD), where the maximum width, $\hbox {SFD}_W$ is indicated.

The axial component of the magnetization dependent interaction field $\alpha _z$ is measured directly from the IRM and DCD remanence curves using,

12 $$\begin{aligned} \alpha _z^{E} = 2(H_r^{0.5}-H_d^0) \end{aligned}$$

where $H_r^{0.5}$ is the field value at which the normalized IRM curve is equal to 0.5, while $H_d^0$ is the field value at which the DCD curve is zero as shown in Fig. 7a^35^. In all cases, the remanence curves were normalized to the maximum value of the IRM remanence curve. The measured values are given in Table 1 as $\alpha _z^E$.

For comparison with the experimental ($\alpha _z^E$) values, the theoretical ($\alpha _z^T$) values of the component of the magnetization dependent interaction field, Eq. (1), have been calculated for each sample using $M_s$(Ni) =485, $M_s$(FeNi) =788, $M_s$(Co) =1400 and $M_s$(CoFe) =1900 (expressed in emu/cm$^3$). These theoretical values of the interaction are presented in Table 1 as $\alpha _z^T$.

The switching field distribution (SFD) is obtained as the derivative of the DCD remanence curve with respect to the field. Its maximum width ($\hbox {SFD}_{\mathrm {W}}$), as shown in Fig. 7b, has been measured in all the samples.

Complementary in-field magnetic force microscopy has also been done on very low packing fraction ($P=$ 0.4%) NW arrays, with wire diameter of 50 nm (S6 [Ni], S13 [CoFe]) and 71 nm (S15 [NiFe], and S16 [CoFe]) to determine their switching field distribution, using a procedure reported recently^43^. Briefly, a smooth surface, where all the nanowire tips evenly exposed to the surface, has been obtained by removing the Au and Cr cathode by chemical etching. The NW initially uniformly magnetized in a field *H* = + 2000 Oe along + Oz while the tip magnetized along – Oz. For example, Supplementary Fig. SI6 shows an MFM image of sample [S16] initially magnetized in the + Oz direction. Then, a series of magnetic fields of increasing intensity and opposite to the sample initial saturation field (– Oz) were applied. To perform the MFM scans, the field was removed, and the magnetic remanent state was measured. This is equivalent to the DC remanent demagnetization procedure. By counting the number of wires magnetized in the positive (+ Oz) and negative (– Oz) directions, the number of switched wires at each field increment has been determined, which leads to the switching field distribution.

## Supplementary information

Supplementary Information.
