# Supplementary material for: Relation of the average interaction field with the coercive and interaction field distributions in First order reversal curve diagrams of nanowire arrays
Source: Sci Rep. 2020 Dec 7;10:21396. doi: 10.1038/s41598-020-78279-1 (PMC7721885; doi:10.1038/s41598-020-78279-1)
Supplement: Supplementary file 1 — Supplementary Information. [file 41598_2020_78279_MOESM1_ESM.pdf]

Relation of the average interaction field with the coercive and interaction field distributions in First order reversal curve diagrams of nanowire arrays

Y. G. Velázquez[1], A. Lobo Guerrero[2], J. M. Martínez[3], E. Araujo[1], M. R. Tabasum[4], B. Nysten[4], L. Piraux[4], and A. Encinas[5,*]

[1]Departamento de Matemáticas y Física, Instituto Tecnológico y de Estudios Superiores de Occidente, Periférico Sur Manuel Gómez Morín 8585, 45604 Tlaquepaque, Jalisco, Mexico
[2]Área Académica de Ciencias de la Tierra y Materiales, Universidad Autónoma del Estado de Hidalgo, Carretera Pachuca Tulancingo km 4.5, Ciudad del Conocimiento, Mineral de la Reforma, Hidalgo, Mexico
[3]Escuela de Ingenieria y Ciencias, Tecnologico de Monterrey, Puebla, Atlixcáyotl 5718, Reserva Territorial Atlixcáyotl, 72453 Puebla, Pue. Mexico
[4]Institute of Condensed Matter and Nanosciences, Université Catholique de Louvain, Place Croix du Sud 1, B-1348, Louvain-la-Neuve, Belgium
[5]División de Materiales Avanzados, Instituto Potosino de Investigación Científica y Tecnológica, Camino a la Presa de San José 2055, 78216 San Luis Potosí, SLP, Mexico
[*]armando.encinas@ipicyt.edu.mx

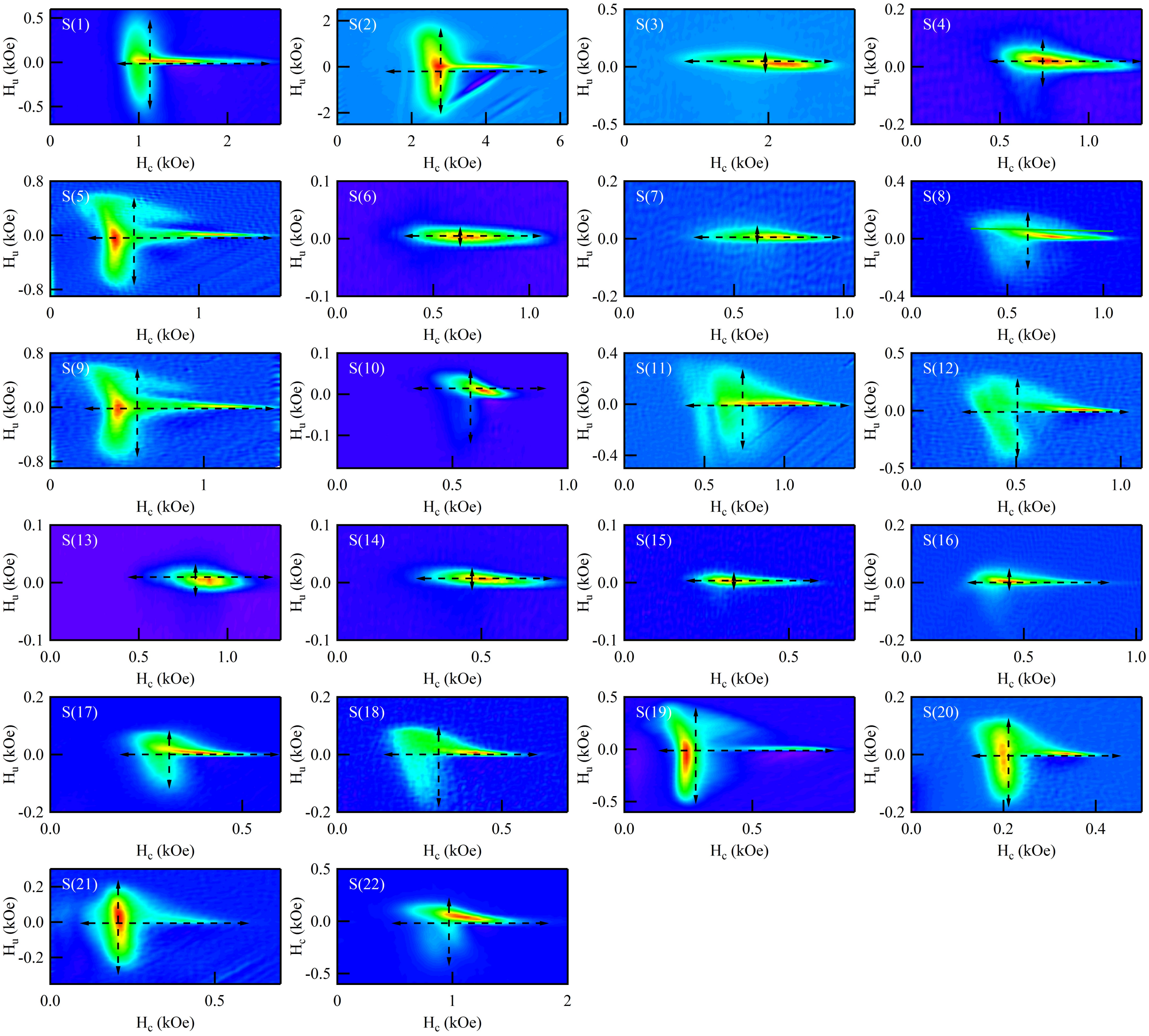

Figure SI1. FORC diagrams for the 22 samples considered in this study plotted in the H_c and H_u plane, where the measured quantities are indicated: the interaction field distribution Δ_{IFD} and the coercive field distribution Δ_{CFD}.

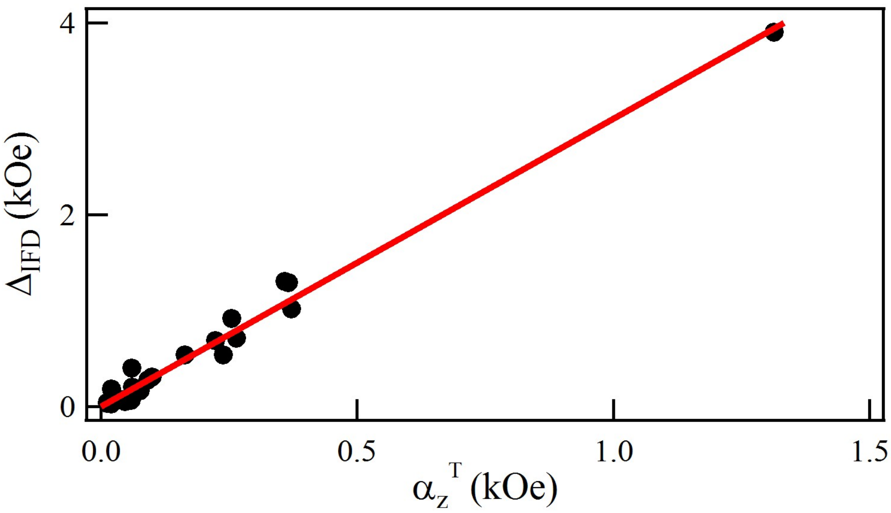

Figure SI2. Measured width of the IFD distribution, Δ_{IFD}, as a function of the axial component of the magnetization dependent average interaction field α_z^T. The straight line corresponds to the fit $\Delta_{IFD}=3\alpha_z$.

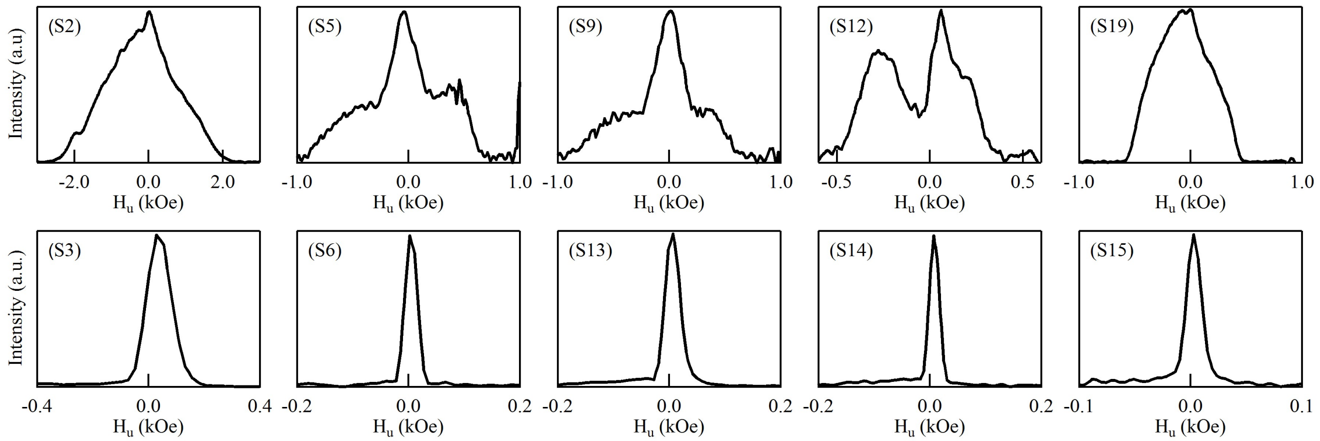

Figure SI3. Interaction field distribution profile of samples with high packing fractions (S2, S5, S9, S12 and S19) and low packing fractions (S3, S6, S13, S14, S15).

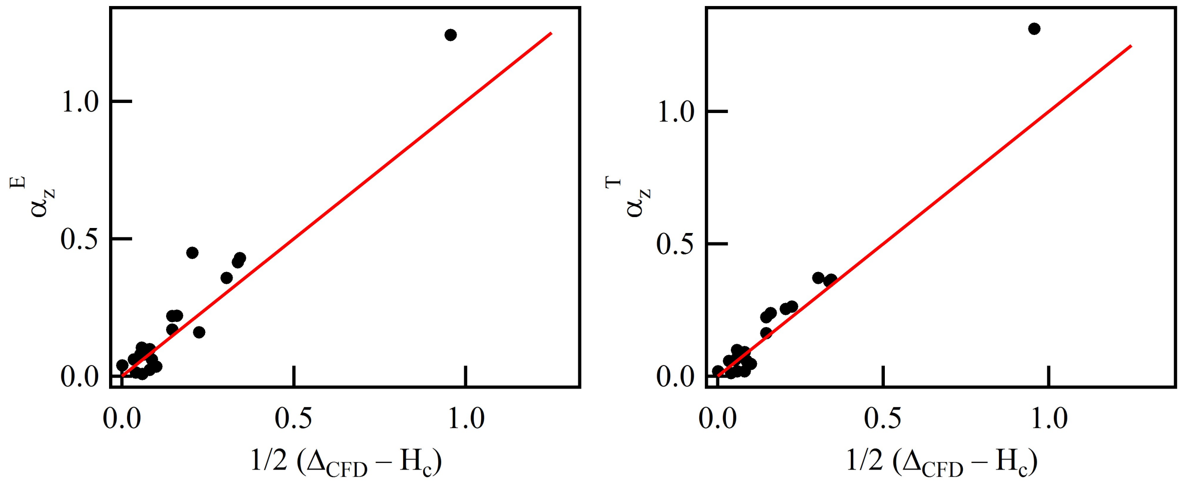

Figure SI4. Comparison of the experimental (left) and theoretical (right) values of the axial components of the magnetization dependent interaction field strength as a function of the Δ_{CFD} measured from the FORC diagram and H_c measured from the major hysteresis loops.

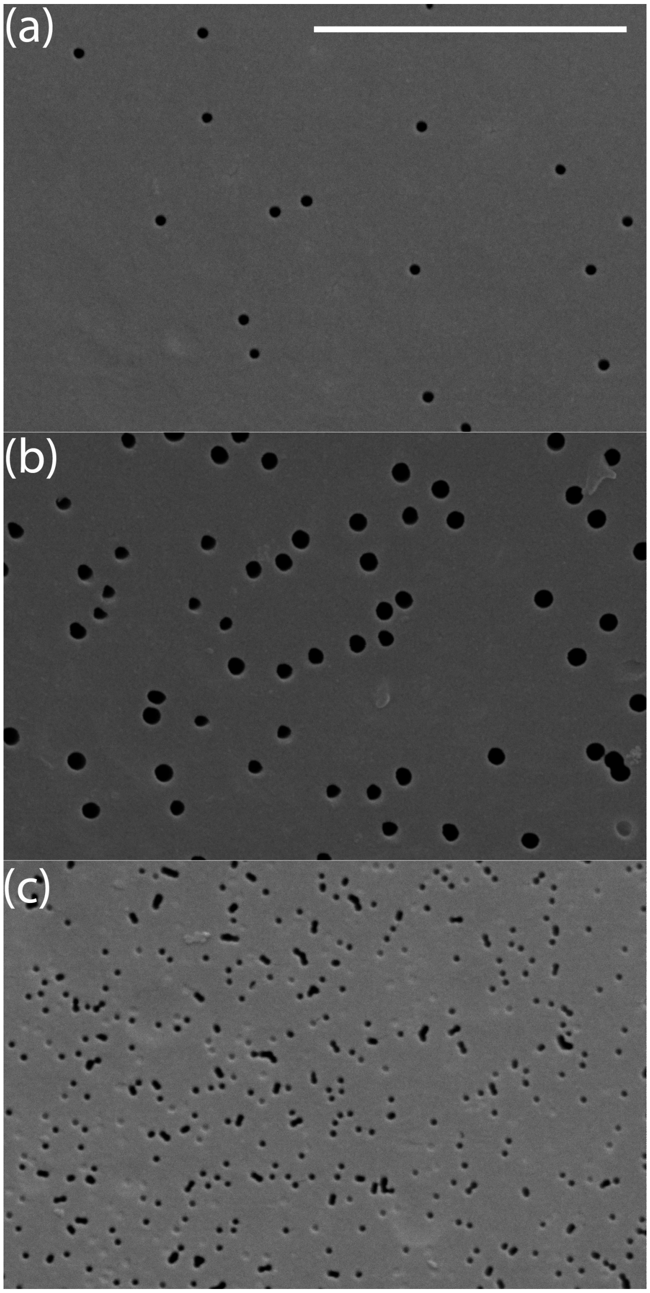

Figure SI5. Top view SEM micrographs of three PC membranes. One (a) with the lowest porosity used, [S14] with 71 nm diameter and 0.4% porosity and two with high porosities (b) [S19] with 100 nm diameter and 10% porosity and (c) [S9] with 50 nm diameter and 11.8% porosity. Scale bar 2μm.

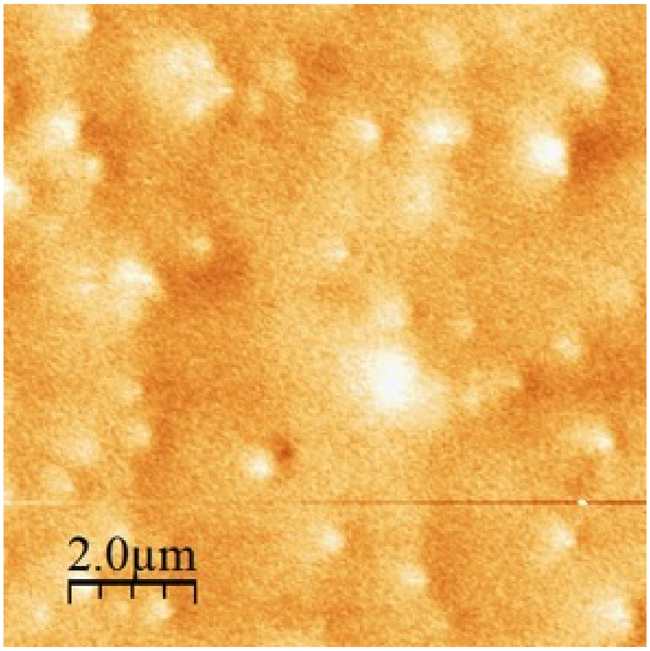

Figure SI6. Magnetic force microscopy image of sample [S16] corresponding to a CoFe nanowire array with 71 nm diameter and a packing fraction (membrane porosity) of $P = 0.4\%$, initially magnetized in the +Oz direction.
